# Two novel mutations of *COMP* in Japanese boys with pseudoachondroplasia

**DOI:** 10.1038/s41439-018-0012-z

**Published:** 2018-06-08

**Authors:** Yosuke Ichihashi, Masaki Takagi, Tomohiro Ishii, Kenji Watanabe, Gen Nishimura, Tomonobu Hasegawa

**Affiliations:** 1 Department of Pediatrics, Keio University School of Medicine, Tokyo, Japan; 2 Department of Clinical Research, Tokyo Metropolitan Children’s Medical Center, Tokyo, Japan; 3 Department of Pediatrics, Kagoshima City Hospital, Kagoshima, Japan; 4 Intractable Disease Center, Saitama Medical University Hospital, Saitama, Japan

## Abstract

Mutations in the cartilage oligomeric matrix protein (*COMP*) gene cause both pseudoachondroplasia (PSACH) and multiple epiphyseal dysplasia (MED). Most mutations in *COMP* are located in the region encoding type 3 thrombospondin like domain (TSP3D). We report two Japanese boys with PSACH who had different novel in-frame deletions in TSP3D. The result recapitulates previous reports in that the in-frame deletions in TSP3D preferentially caused PSACH rather than MED.

Pseudoachondroplasia (PSACH; MIM# 177170) is a rare autosomal dominant chondrodysplasia characterized by specific clinical features, such as disproportionately short stature, brachydactyly, loose joints, ligamentous laxity, and early onset osteoarthritis^1–3^. Mutations in the cartilage oligomeric matrix protein (*COMP*) gene give rise to a spectrum of disorders, severe PSACH, and mild multiple epiphyseal dysplasia (MED)^4^. COMP is a calcium-binding homopentameric protein that forms disulfide bonds between monomers^5^. The COMP monomer contains eight type-3 thrombospondin like domains (TSP3Ds, T1 to T8)^6^, which is where approximately 85% of the *COMP* mutations were identified^7^. Each TSP3D has a consensus motif, where one or two calcium ion-binding consensus sequences (DXDXDGXXDXXD) reside. The number of binding calcium ions alters the conformation and function of the COMP protein^8^. Briggs et al.^7^ reported that missense mutations in T6, T7, and T8 and in-frame deletions in TSP3D preferentially cause PSACH rather than MED. We report two children with PSACH who each had a novel in-frame deletion.

## Case 1

The proband visited our outpatient clinic at 6 years and 2 months of age. He was born to non-consanguineous Japanese parents at 40 weeks of gestation. The pregnancy and delivery were uncomplicated. His birth weight, length, and head circumference were 3570 g ( + 1.43 SD), 49.7 cm (+0.33 SD), and 34.8 cm (+0.96 SD), respectively. There was no family history of short stature or any skeletal diseases.

**Fig. 1:**
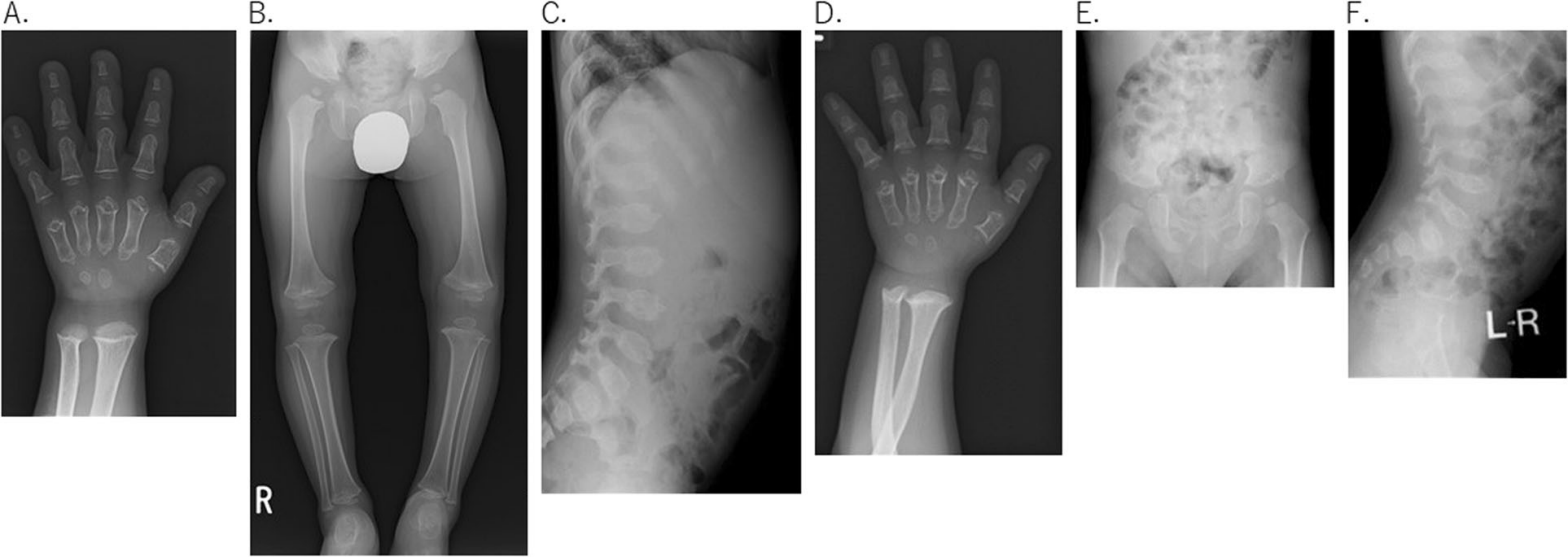
Radiographic findings of two boys with PSACH. **a**, **b**, and **c** show X-ray images in case 1, and **d**, **e**, and **f** show X-ray images in case 2

Growth retardation was evident at 2 years of age. At 4 years, he was diagnosed with PSACH due to waddling gait, short limbs, and radiologic features, including oval-shaped platyspondyly, anterior breaking of vertebrae, small and irregular epiphyses, and irregular metaphyses (Fig. 1a, b, c).

## Case 2

The proband sought medical attention at 2 years and 9 months of age. He was born to non-consanguineous Japanese parents via vaginal delivery at 39 weeks and 1 day of gestation. The pregnancy and delivery were uncomplicated. His birth weight, length, and head circumference were 3274 g (+0.49 SD), 49.5 cm (+0.34 SD), and 34.0 cm (+0.59 SD), respectively. There was no family history of short stature or any skeletal diseases.

He attained developmental milestones normally until 1 year of age. He presented initially with gait disturbance at 1 year and 9 months of age. He visited an orthopedic clinic, and metaphyseal irregularity and epiphyseal hypoplasia of the femur and tibia were noted. He was diagnosed as having PSACH based on his short stature (−2.18 SD), waddling gait, and radiologic findings, such as oval-shaped platyspondyly, anterior breaking of vertebrae, small and irregular epiphyses, and irregular metaphyses (Fig. 1d, e, f).

Genomic DNA was extracted from peripheral white blood cells. We examined all coding exons and flanking introns of the *COMP* gene by Sanger sequencing. Primer sequences and PCR conditions are available upon request. This study was approved by the Ethics Committee of Keio University School of Medicine or Ethics Committee of Tokyo Metropolitan Children’s Medical Center. We obtained written informed consent for molecular studies from the parents. We assessed the pathogenicity of genomic variations of *COMP* using University of California Santa Cruz (UCSC) Genome Browser for evolutionary conservation, Human Gene Mutation Database (HGMD) for previous descriptions, and the Exome Aggregation Consortium (ExAC) database for allele frequency.

**Fig. 2:**
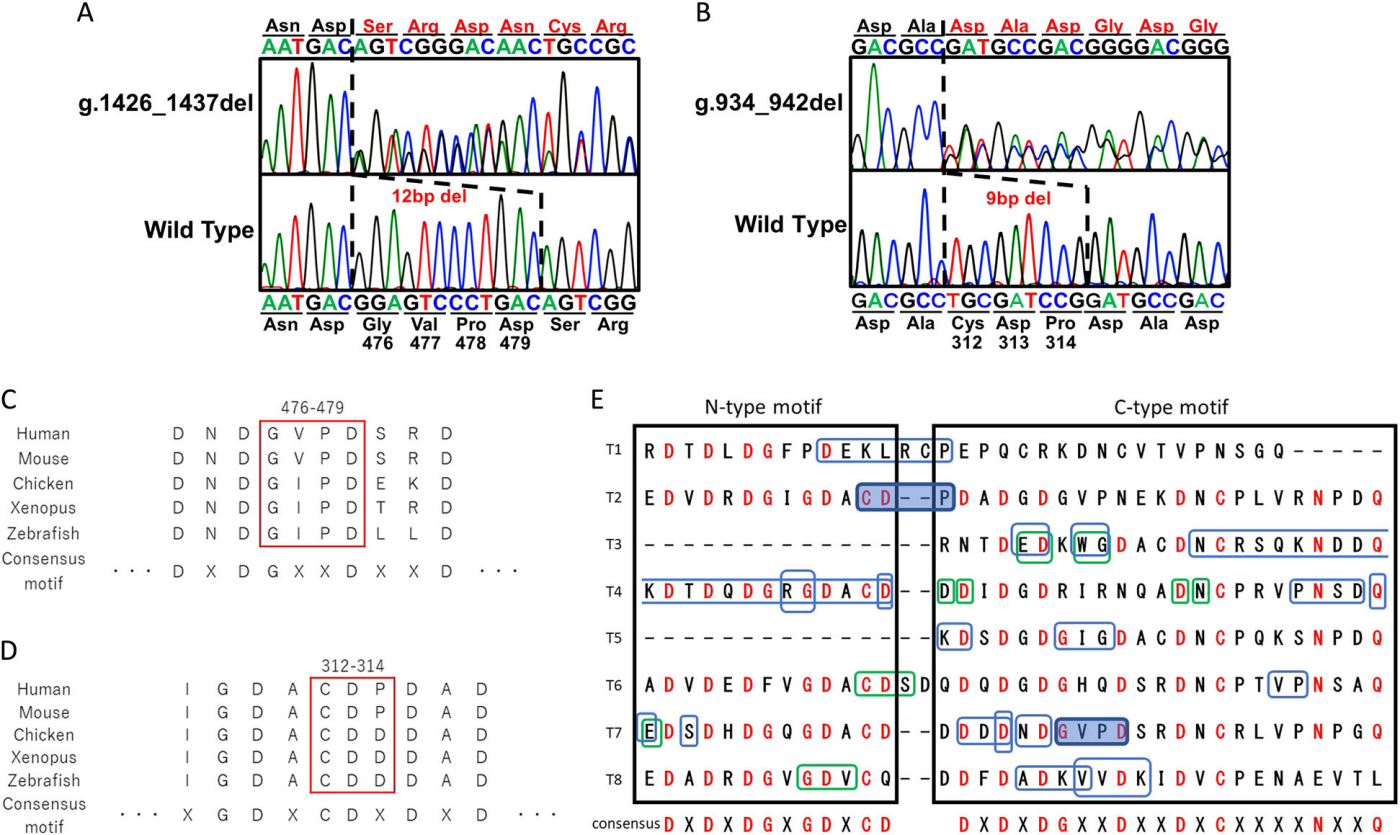
Genetic analysis of the COMP gene. **a** Chromatogram of exon 13 in case 1 indicates a 12-base deletion. **b** Chromatogram of exon 9 in case 2 indicates a 9-base deletion. **c** Evolutionarily conserved amino acids around the in-flame deletion in case 1. The red box shows deleted amino acids. **d** Evolutionarily conserved amino acids around the in-flame deletion in case 2. The red box shows deleted amino acids. **e** Consensus amino acid sequence of the type-3 TSP like domain. T1 to T8 represents each repeat. Each amino acid of the consensus motif is shown in red. The blue boxes show in-frame deletions in PSACH, and the green boxes show in-frame deletions in MED. The novel in-frame deletions in our patients are shaded in blue

We identified a heterozygous in-frame deletion in each case: a 12-base deletion in exon 13 (NM_000095(COMP_v001):c.1426_1437del [p.Gly476_Asp479del]) for case 1 (Fig. 2a) and a 9-base deletion in exon 9 (NM_000095(COMP_v001):c.934_942del [p.Cys312_Pro314del]) for case 2 (Fig. 2b). In both cases, the parents did not provide consent for further genetic analysis of family members. Both deleted amino acids are evolutionarily conserved (Fig. 2c, d) and are located on TSP3D, resulting in a partial loss of the consensus motif of TSP3D (Fig. 2e). Neither deletion was recorded in either HGMD or the ExAC database.

We reported two novel in-frame deletions in the *COMP* gene in PSACH children. Unfortunately, we were not able to ascertain that these deletions were de novo. However, it is very likely that both deletions are pathogenic because the deleted segments spanned amino acid residues that are conserved among different species and are compatible with the consensus motif in TSP3D.

The deleted amino acids in case 1 reside in T7, which is a common deletion for PSACH (p.Asp469del). The common deletion removes a single amino acid in the calcium ion-binding consensus sequence. The biological consequence must be low affinity for calcium ion binding because the conformation of the COMP protein depends on the number of bound calcium ions^8^. Likewise, it is tempting to assume that the removal of two amino acids in the calcium ion-binding consensus sequence due to the deletion in case 1 conspicuously impaired calcium ion affinity. The deleted amino acids in case 2 include cysteine 312, which is known to form a disulfide bond with cysteine 292^9^; thus, the mutant protein creates a severe conformational change.

This study reported two novel pathogenic in-frame deletions in the *COMP* gene and confirmed the previous notion that in-frame deletion preferentially causes PSACH rather than MED.

### HGV database

The relevant data from this Data Report are hosted at the Human Genome Variation Database at 10.6084/m9.figshare.hgv.2318 and 10.6084/m9.figshare.hgv.2321.
